# A New Periodical

**Published:** 1879-06

**Authors:** 


### A NEW PERIODICAL.

The first number of "The Dental Luminary," a quarto of twelve pages, edited and published by Drs. J. P. and W. P. Holmes, Macon, Ga., has just made its appearance. It is devoted to matters of interest to the dental profession.

This number contains much good matter and gives evidence of life and activity that promises well for the future. It follows in the wake of the "Office and Laboratory" and "Dental News," and assumes the quarto form. This is a very inconvenient shape for such a work. It occupies too much space unless folded upon itself, and that mutilates it. It can not be stitched and cut as the octavo form, and in addition to this, being so nearly the ordinary newspaper form it is almost certain to be destroyed. These quartos would each be more valuable if they would change the size. Drs Holmes are engaged in the manufacture and sale of dental goods, as well as practice ; and if they do the former as well as they can the latter, the dental profession of the South will have occasion for thankfulness.

